# Impaired Phagocytosis in Localized Aggressive Periodontitis: Rescue by Resolvin E1

**DOI:** 10.1371/journal.pone.0024422

**Published:** 2011-09-14

**Authors:** Gabrielle Fredman, Sungwhan F. Oh, Srinivas Ayilavarapu, Hatice Hasturk, Charles N. Serhan, Thomas E. Van Dyke

**Affiliations:** 1 Department of Periodontology, The Forsyth Institute, Cambridge, Massachusetts, United States of America; 2 Department of Anesthesiology, Perioperative, and Pain Medicine, Center for Experimental Therapeutics and Reperfusion Injury, Brigham and Women's Hospital Harvard Medical School, Boston, Massachusetts, United States of America; Ludwig-Maximilians-Universität München, Germany

## Abstract

Resolution of inflammation is an active temporally orchestrated process demonstrated by the biosynthesis of novel proresolving mediators. Dysregulation of resolution pathways may underlie prevalent human inflammatory diseases such as cardiovascular diseases and periodontitis. Localized Aggressive Periodontitis (LAP) is an early onset, rapidly progressing form of inflammatory periodontal disease. Here, we report increased surface P-selectin on circulating LAP platelets, and elevated integrin (CD18) surface expression on neutrophils and monocytes compared to healthy, asymptomatic controls. Significantly more platelet-neutrophil and platelet-monocyte aggregates were identified in circulating whole blood of LAP patients compared with asymptomatic controls. LAP whole blood generates increased pro-inflammatory LTB4 with addition of divalent cation ionophore A23187 (5 µM) and significantly less, 15-HETE, 12-HETE, 14-HDHA, and lipoxin A_4_. Macrophages from LAP subjects exhibit reduced phagocytosis. The pro-resolving lipid mediator, Resolvin E1 (0.1–100 nM), rescues the impaired phagocytic activity in LAP macrophages. These abnormalities suggest compromised resolution pathways, which may contribute to persistent inflammation resulting in establishment of a chronic inflammatory lesion and periodontal disease progression.

## Introduction

Periodontitis and other periodontal diseases (PD) comprise a unique and complex group of inflammatory conditions that result in the destruction of the supporting structures of the dentition [1]. PD is a chronic inflammatory disease initiated by bacterial biofilms that naturally form on the teeth that is associated with, and is thought to exacerbate, the symptoms of several inflammatory disorders such as arthritis, Type II diabetes, preeclampsia, conditions associated with preterm low birth weight, and cardiovascular diseases (CVD) [2], [3], [4], [5], [6]. PD is a major public health concern given that it is among the most prevalent human diseases [7]. Since the pathogenesis of PD has strikingly similar aspects to many other inflammatory diseases, it has become a recognized model for examining effector cell mediated inflammation [8].

The etiology of PD is bacterial plaque and specific Gram-negative micro-organisms, such as *Porphyromonas gingivalis* and *Tannerella forsythensis* in the case of chronic periodontitis, and *Aggregatobacter actinomycetemcomitans* in the case of Localized Aggressive Periodontitis, are associated with the subgingival biofilm in disease. [1], [9]. The bacteria are necessary, but not always sufficient to produce disease [10] expression of disease is associated with modifiable risk factors such as smoking and genetic risk factors such as the inflammatory response. PD progresses in periodic, relatively short episodes of rapid tissue destruction followed by some repair, and prolonged intervening periods of disease remission [11]. Despite the apparent stochastic distribution of episodes of disease activity, the resulting tissue breakdown results in alveolar bone loss and pocket formation, which is common to several forms of PD. While LAP is clinically distinct from other types of periodontitis, it seems to represent the extreme with regard to inflammatory abnormalities. Chronic periodontitis is also associated with impaired phagocytosis [12] as well as other hyper-inflammatory traits. LAP is characterized by functional abnormalities of host cells, particularly neutrophils [13], [14] that possess a hyper-activated or primed phenotype [1], [15]. The functional consequences of neutrophil priming include dysregulated chemotaxis, phagocytic abnormalities, and heightened pro-inflammatory activity including increased oxidative stress and secretion of inflammatory mediators [16], [17], [18]. Hence, LAP PMN hyperfunction yields its inability to clear bacteria resulting in tissue damage and chronic lesions [1]. Of note, the cells used in several of these reports are circulating PMN, not just of those within the inflammatory milieu. Hyperactive circulating PMN indicates an underlying systemic component to this disease, suggesting persistent inflammation that does not resolve.

Resolution of inflammation is crucial for tissue homeostasis and necessary for ongoing health [19]. Resolution programs require the local biosynthesis of endogenous specialized pro-resolving lipid mediators (SPMs). These SPM include the lipoxins, resolvins, protectins and maresins [19], which are enzymatically synthesized via sequential steps involving lipoxygenases (LOX), and cyclooxygenases (COX). Arachidonic acid (AA) derived Lipoxin A_4_ (LXA_4_), as an example, is generated through two distinct transcellular pathways; a 15-LOX and 5-LOX biosynthetic pathway or a 5-LOX and 12-LOX pathway [20], [21]. Cell: cell interactions determine the source of the LOX isoforms. The lipoxygenases are critical enzymes for the formation of LXA_4_ as well as the omega-3 EPA and DHA derived resolvins, protectins are maresins [19]. SPM are dual functioning because they limit neutrophil accumulation and stimulate non-phlogistic activation of macrophages *in vivo* [19], [22]. SPM have actions on selective cellular targets and act via specific G-protein coupled receptors [19]. It is noteworthy that EPA-derived Resolvin E1 (RvE1) is protective in several inflammatory disease models, including experimental periodontitis [8].

Recent evidence suggests that a failure in mounting endogenous resolution programs may be a key feature in various inflammatory disorders such as atherosclerosis [23], [24]. The mechanisms underlying failed resolution are not known, but increasing evidence suggests an imbalance between pro- inflammatory and pro-resolving mediators to be a factor [25]. Here, we present an example of a human disease that exhibits a pro-inflammatory cellular phenotype and a malfunction in the capacity to generate LXA_4_ and SPM precursors. LAP has activated circulating platelets and leukocytes and increased platelet-leukocyte aggregates compared to healthy, asymptomatic controls. There is also an imbalance in the release pro-inflammatory and anti-inflammatory LOX-derived lipid mediators from stimulated whole blood of LAP. Additionally, phagocytosis of opsonized zymosan particles by LAP macrophages was markedly reduced compared to healthy controls and RvE1 rescued the impairment. Hence, these results with LAP suggest an inability to effectively mount resolution pathways contributing to persistent, non-resolving, chronic lesion and periodontal disease progression.

## Results

### LAP has increased platelet–leukocyte aggregates in whole blood

**Figure 1:**
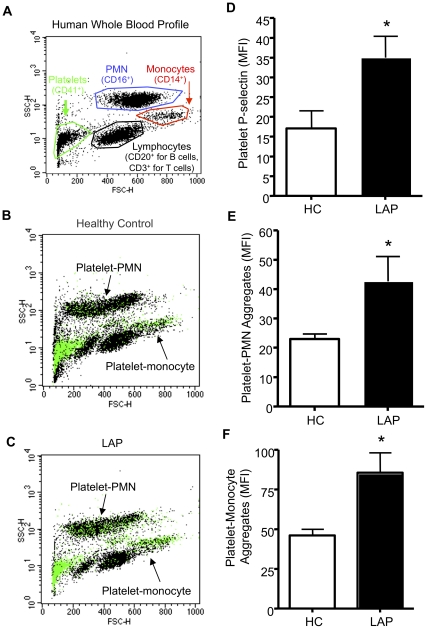
LAP platelets and leukocytes in whole blood. Venous whole blood was collected from LAP or healthy control donors. (A) Platelets, PMN or monocytes were characterized based on positive staining of cell specific antibodies (CD41, CD16, CD14, respectively) and characteristic cell morphology (representative dot plot). (B,C) Representative dot plots of platelet-leukocyte aggregates. Green spots indicate CD41^+^ platelets. (D) CD41^+^CD62P^+^ were assessed and quantified via flow cytometry and Cell Quest software (LAP platelets, black bar; healthy control, white bar). (E) PMN or (F) monocyte populations with CD41^+^ staining. Results are mean ± SEM, n = 4, *p<0.05.

Platelets, like neutrophils, are key cellular mediators of innate immune responses [26]. It is known that neutrophils from LAP patients are primed [27], thus it was of interest to assess whether platelets in LAP blood are hyperactive as well. Whole blood from healthy control volunteers or patients diagnosed with LAP was characterized based on cellular morphology and select antibody staining (Figure 1A–C). In unstimulated whole blood, platelet P-selectin was significantly increased (∼50%) on the surface of LAP platelets as compared to healthy control (Figure 1D).

Aberrant formation of platelet-neutrophil and platelet-monocyte aggregates is associated with inflammatory diseases, such as cardiovascular diseases [28] and chronic periodontitis [29]. Since P-selectin is increased on LAP platelets, it was of interest to monitor whether whole blood from LAP donors exhibited increased platelet-leukocyte aggregates as compared to healthy control. LAP exhibited significant increases in platelet-PMN and platelet-monocyte (∼50% and ∼30% respectively) aggregates (Figure 1B, E, and C, F respectively) compared to healthy control. Representative dot plots demonstrate platelet-leukocyte aggregates where platelets (CD41^+^) are represented as green spots from healthy control (Figure 1B) or LAP (Figure 1C).

**Figure 2:**
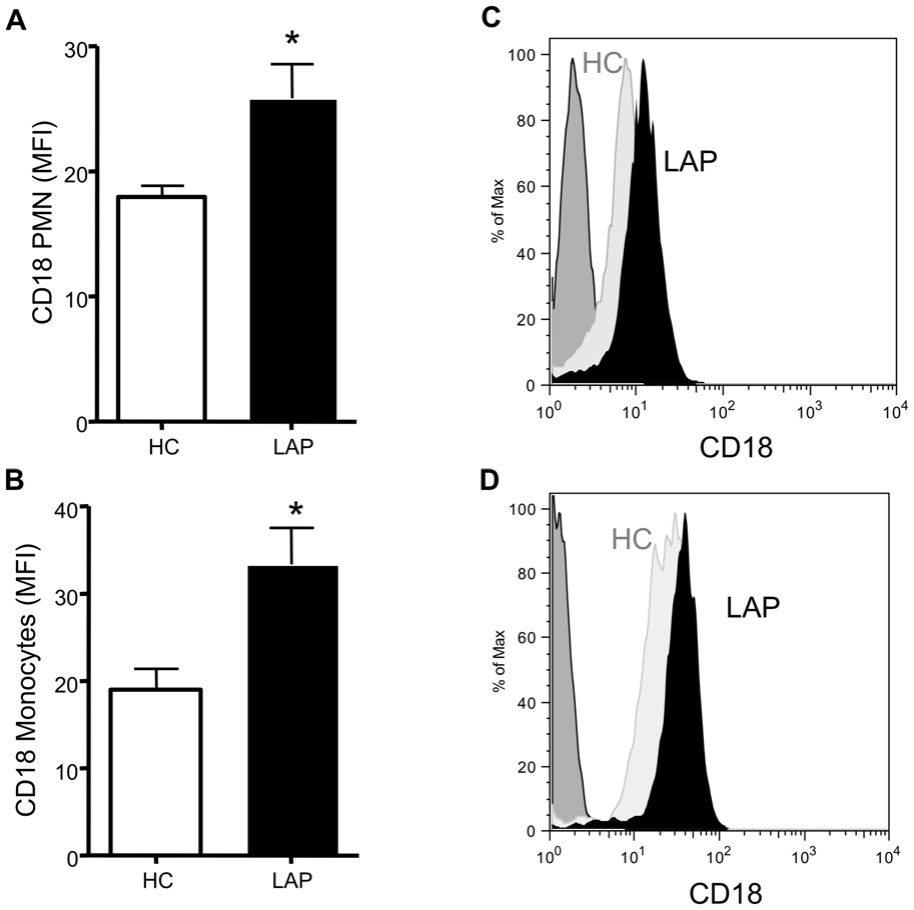
LAP patients display activated leukocytes in whole blood as compared to healthy control. CD18 was monitored on (A) PMN or (B) monocytes in whole blood without the addition of exogenous stimuli from healthy control (white) versus LAP (black). Results are mean ± SEM, n = 4, *p<0.05. Representative histograms of CD18 surface expression on (C) PMN and (D) monocytes. HC, healthy control, light grey; LAP, solid black; IgG dark grey.

Leukocyte recruitment to inflamed areas requires a precise sequence of events that initially involves the interaction of platelets, leukocytes and activated endothelial cells via selectins and integrins [26]. Therefore, here we assessed integrin surface expression on leukocytes as a marker for activated of leukocytes. LAP exhibited higher surface expression of CD18 on PMN (Figure 2A, C) and monocytes (Figure 2B, D) as compared to healthy control. Representative histograms demonstrate that LAP (black) has a higher surface expression of CD18 on PMN (Figure 2C) and monocytes (Figure 2D) as compared to healthy controls (light gray). Together, these results indicate that LAP resting cells within whole blood are circulating in a hyperactive or primed state.

### Imbalance in LOX biosynthetic markers in LAP

**Figure 3:**
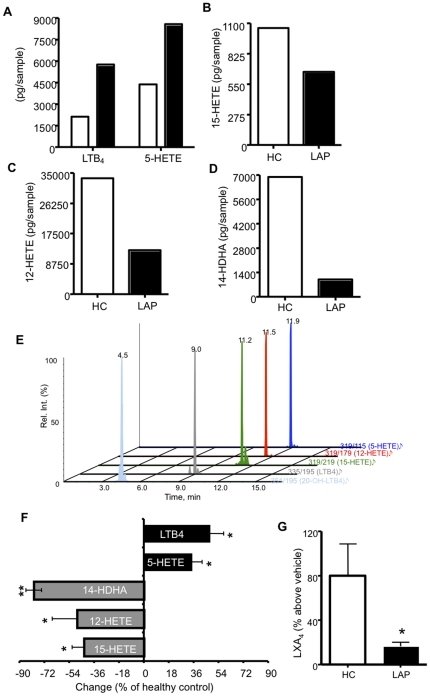
LAP whole blood displays Increased capacity for 5-HETE and LTB_4_ levels, and decreased for 12-HETE, 14-HDHA, 15-HETE and LXA_4_. Whole blood was stimulated A23187 (5 µM) for 20 minutes, 37°C. Incubations were stopped on ice and plasma was collected for LC-MS/MS or ELISA analysis. (A–D) Representative LC-MS/MS quantitation of A23187-stimulated whole blood from healthy control (HC, white bars) and LAP (black bars) pairs. (E) Representative chromatogram. (F) Percent change of indicated lipid mediators. Results are meant ± SEM n = 3, *p<0.05, **p<0.01. (G) Percent increase compared to vehicle of LXA_4_ was analyzed by ELISA. Results are mean ± SEM, n = 10, *p≤0.05, healthy control versus LAP.

LTB_4_ and other pro-inflammatory mediators were detected in the gingival crevicular fluid (GCF) of chronic periodontitis [30] and LAP patients [31]. We next questioned the pro-inflammatory and pro-resolving mediator formation within whole blood. To investigate the capacity of whole blood cells to generate and release lipid mediators, whole blood was stimulated with A23187 (5 µM) or vehicle, plasma was collected, subjected to solid-phase extraction followed by LC-MS/MS analysis (Table 1). Representative donor pairs demonstrated increased LTB_4_ and 5-HETE (Figure 3A), and decreased 15-HETE (Figure 3B), 12-HETE (Figure 3C) and 14-HDHA (Figure 3D) levels plasma obtained from whole blood of LAP patients with the addition of A23187 (*ex vivo*) as compared to healthy controls (Table 1). A representative chromatogram is shown in Figure 3E. LAP, compared to its age, gender and race matched asymptomatic controls, has increased 5-LOX products including LTB_4_ (47.7%±9.8) and 5-HETE (34.6%±7.2) indicating a hyperactive phenotype (Figure 3F). Of note, 12-LOX and 15-LOX products including 12HETE, 14HDHA and 15-HETE (48.5%±17.9, 79.8%±5.6, and 43.5%±8.5, respectively) were all significantly lower in stimulated LAP plasma compared to healthy controls (Figure 3F).

**Table 1:** LAP whole blood LOX capacity versus healthy donors.

Donor Pairs	LTB_4_	20-OH LTB_4_	5-HETE	15-HETE	12-HETE	14-HDHA
Healthy control	1205.0	393.5	1335.0	365.5	3280.0	2273.6
LAP	2432.9	363.6	1878.3	246.4	2851.5	326.6
Healthy control	2120.4	682.9	4367.4	1057.4	33430.6	6903.4
LAP	5760.6	1191.8	8584.2	660.4	12699.0	107.1
Healthy control	1761.5	369.3	2407.4	839.6	17615.2	1361.1
LAP	2498.8	489.9	3253.3	332.1	5193.6	427.7

*Whole blood (1 mL) was incubated with A23187 (5 µM) for 20 mins, 37°C. Incubations were stopped on ice and plasma was collected for C-18 solid phase extraction and subjected to LC-MS/MS based lipidomics. Values are represented as pg/sample.

Since there is aberrant production of 5-,12-,and 15-LOX products, it was of interest to investigate the pro-resolving transcellular biosynthesis product, LXA_4_. There was significantly less LXA_4_ generation (375 pg/mL in healthy controls, 242 pg/mL in LAP) in A23187-stimulated whole blood of LAP donors (Figure 3G). These results suggest an imbalance between pro-inflammatory and pro-resolving mediator generation.

### RvE1 Rescues Impaired Phagocytosis in LAP macrophages

**Figure 4:**
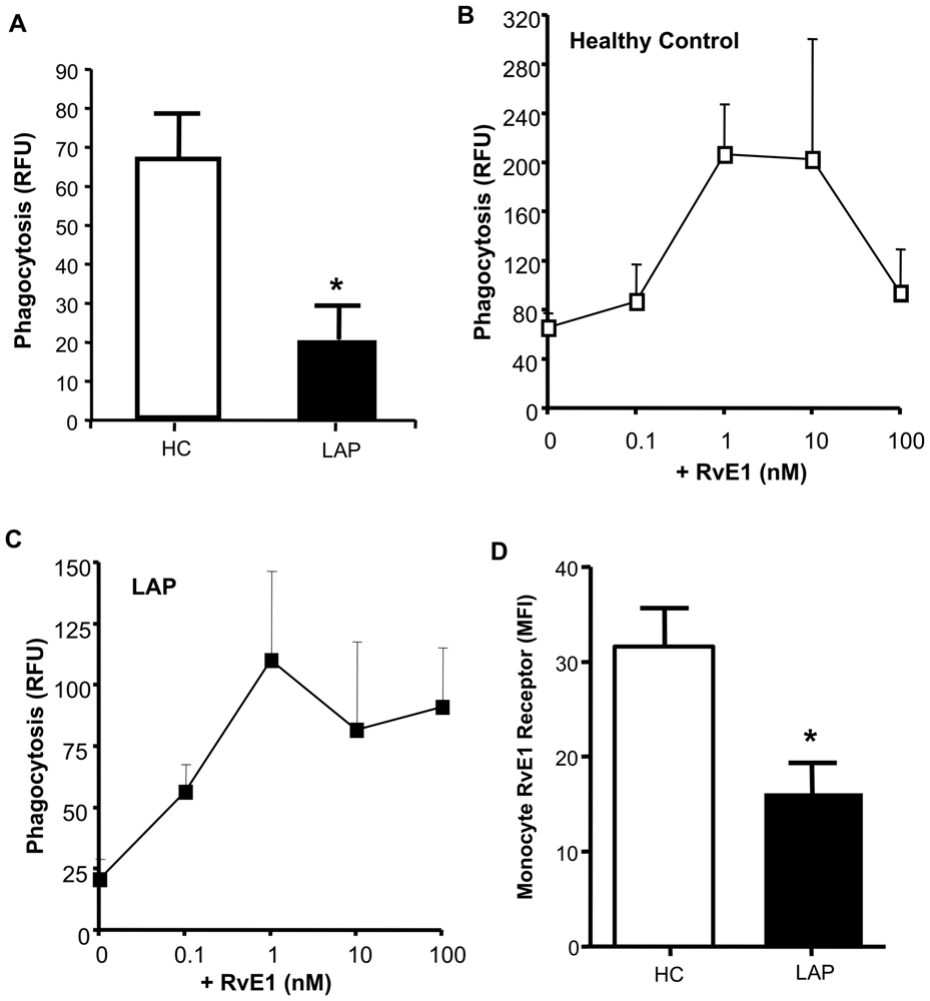
LAP Macrophages display impaired phagocytosis of opsonized zymosan particles. Monocytes were collected from venous whole blood and differentiated into macrophages in the presence of GMCSF (10 ng/mL) for 7 days. After differentiation macrophages were incubated with vehicle or RvE1 (0.1–100 nM) for 15 minutes. Phagocytosis was carried out for 30 minutes, 37°C and analyzed by a Victor3 fluorescent plate reader. (A) Healthy control (white bar) and LAP (black bar) baseline phagocytosis. RvE1 (0.1–100 nM) dose response, (B) HC (C) LAP. (D) Monocytes in whole blood were stained with anti-human ChemR23 and analyzed by flow cytometry. Results are mean ± SEM, n = 4, *p<0.05.

Host defense mechanisms are abnormal in LAP [32]. Macrophages are essential for host defense because of their capacity to clear cellular debris and pathogens for the eventual return to homeostasis. LAP macrophage phagocyte function has not been investigated; therefore, it was of interest to determine whether LAP has normal phagocytic activity as compared to healthy control. There were no differences in viability of LAP and healthy control macrophages after isolation and Wright-Giemsa staining revealed no apparent difference in morphology between LAP and healthy control macrophages. Figure 4A demonstrates that LAP macrophages are impaired at phagocytizing serum treated FITC-zymosan (SZ). Since RvE1 is known to stimulate macrophages to enhance phagocytosis [22], [33], it was of interest to investigate whether RvE1 would rescue the phagocytic defect observed in LAP. When incubated 15 minutes prior to SZ addition, RvE1 enhanced phagocytosis in healthy control (Figure 4B) and LAP macrophages (Figure 4C). Of note, RvE1 as low as 1 nM restored phagocytic activity of LAP macrophages to levels comparable to healthy controls (Figure 4C). ChemR23, an RvE1 receptor, has lower surface expression on circulating monocytes in whole blood (Figure 4 D). These results indicate that although LAP has impaired phagocytosis, RvE1, when added exogenously, can rescue the phagocytic defect.

## Discussion

Periodontal diseases, such as LAP, result in the inflammatory destruction of the supporting tissues of the dentition. While the etiology of periodontitis is bacterial, it is becoming clear that the pathogenesis of disease is mediated by the host response [34]. Given the essential role of the innate immune system in regulating immunity, it is conceivable that dysfunction of the components of resolution can contribute to disease [35]. Hence, it was of interest to investigate whether distinct cellular and molecular pathways of resolution from LAP subjects were aberrantly regulated. Here, we report that (i) LAP platelets, neutrophils and monocytes in whole blood display increased integrin and selectin surface expression compared to healthy control, as well as increased platelet-neutrophil and platelet-monocyte aggregates, (ii) the generation of LXA_4_ and maresin precursor, 14-HDHA was compromised in LAP whole blood and (iii) macrophages from LAP patients exhibit impaired phagocytosis as compared to healthy control.

Inflammation, a common feature of periodontal and cardiovascular diseases [36], [37], can be assessed at the cellular level by investigating the interactions between platelets and leukocytes. Figure 1 displays significantly elevated levels of P-selectin on platelets as well as increased platelet-leukocyte aggregates in LAP subject blood compared to healthy control blood. Since P-selectin is mobilized to the platelet surface upon activation, our results suggest that LAP platelets are circulating in a hyperactive state. In addition, platelets and leukocytes generally aggregate during inflammation or in a pathological milieu, such as those associated with CVD. In fact, circulating monocyte-platelet aggregates have been reported to be an early marker of myocardial infarction [28]. Therefore, the hyperactive state of LAP platelets as well as their increased association with leukocytes provides a further mechanistic link between periodontal and cardiovascular diseases [37].

To corroborate our finding that leukocytes are circulating in an activated state, we also profiled pro-inflammatory chemical mediators. LAP blood produced increased 5-LO products such as 5-HETE and LTB_4_ (Figure 3), which is consistent with earlier reports that demonstrate LTB_4_ within inflammatory periodontal exudates. In addition to periodontal disease, elevated LTB_4_ levels have also been associated with other non-communicable chronic inflammatory disease such as atherosclerosis [38]. Patients with atherosclerosis were found to possess a 5-LO gene variant leading to increased LTB_4_ especially when on an omega-6 rich diet [39]. Of note, when these patients were placed on an omega-3 diet, there was significantly less LTB_4_ generation [39].

12- and 15-LOX released products were decreased in LAP stimulated blood as compared to healthy controls (Figure 3). Of relevance, in a rabbit model of experimental periodontitis, overexpression of 15-LOX was protective against *P. gingivalis* induced bone loss [40]. Importantly, these rabbits were also resistant to CVD. The protective role of these lipoxygenase-derived products extends beyond that of periodontal disease models. In a pre-clinical disease model of atherosclerosis, 12/15 LOX knockout mice displayed increased atherosclerosis as compared to wild type [23]. 12- and 15- LOX are also critical enzymes for the biosynthesis of lipoxins. LXA_4_ is generated via transcellular biosynthesis between PMN (5-LOX) and platelet (12-LOX) [20], [41] or 15-lipoxygenase (15-LOX) and PMN (5-LOX) [20] interactions. LXA_4_ levels were decreased in LAP compared to healthy control (Figure 3E) most likely due a result of aberrant activation of 12- or 15-LOX. Decreased generation of LXA_4_ was also seen in cystic fibrosis (CF), where CF patients displayed increased platelet-leukocyte aggregates, yet compromised LXA_4_ generation compared to healthy controls [25]. Hence, it is possible that compromised lipoxygenase pathways and the generation of lipoxin or other pro-resolving mediators may be an underlying component of several inflammatory diseases.

Since LAP is an inflammatory disease exacerbated by microbes, functional phagocytes to clear pathogens is of utmost importance for the return to homeostasis. Here, we report that LAP macrophages do not phagocytize opsonized zymosan A as readily as healthy control (Figure 4). The impairment in phagocytosis of opsonized zymosan may be attributed to the known polymorphisms of Fcγ receptors on leukocytes of periodontal disease patients [42]. Omega-3 EPA-derived RvE1 that rescued the phagocytic activity of LAP macrophages (Figure 4) was also shown to enhance efferocytosis *in vitro and in vivo* [22]. RvE1 was also reported to be protective on LAP PMN by dampen ingfMLP-stimulated O_2_^-^ release from LAP PMN [8]. RvE1 is protective for *P. gingivalis* -induced bone loss in experimental periodontitis [8], [43]. Of note, RvE1 is also protective in asthma [44] and acute lung injury [45] models underscoring its role as regulator of several inflammatory diseases. Recent work demonstrated that RvE1 initiates direct activation of the ChemR23 receptor on human macrophages and signals receptor dependent phosphorylation during phagocytosis of opsonized zymosan [46].

Several investigations suggest that bone loss in PD is linked to an imbalance between omega-6 and omega-3 fatty acids [47]. Increased levels of omega-6 AA-derived products, including prostaglandin E_2_ (PGE_2_), thromboxane, prostacyclin and leukotriene B_4_ (LTB_4_) were found in inflamed gingival tissues and gingival crevicular fluid (GCF) [48], [49]. Clinical studies investigating serum polyunsaturated fatty acid (PUFA) levels in periodontal disease patients demonstrated that omega-6 fatty acids were higher in patients with bone loss than in the control group; reduced bone loss was seen in patients with increased serum omega-3 levels [47]. Additionally, daily supplementation of omega-3 fatty acids showed a reduction in periodontal disease gingival pocket formation with an increase of attachment in periodontal disease patients indicating that omega-3 fatty acids are protective against inflammatory bone loss [50].

In addition to environmental factors, there is compelling evidence for genetic components associated with disease progression in LAP [51] (reviewed in [52]). As mentioned earlier LAP PMN exhibit a number of functional abnormalities including impaired chemotaxis, phagocytic abnormalities, and increased ROS generation to name a few [16], [17], [18]. As an example, LAP PMN that display decreased chemotaxis to n-formylated peptides like fMLP can at least in part be explained by a genetic variants associated with FPR receptors [53], [54]. The phenotypic abnormalities presented in this report imply that our patient population may also have a genetic abnormality pertaining to a deficiency in the biosynthesis of lipoxygenase pathways involved in the production of pro-resolving mediators.

Together, these findings demonstrate that cells from LAP patients present a heightened pro-inflammatory phenotype with a compromised capacity to generate SPM. LAP has hyperactive platelets, PMN and monocytes and increased platelet-leukocyte aggregates in the circulation compared to healthy controls. In addition, essential host defense mechanisms, such as removal of pathogens via macrophage phagocytosis are compromised in LAP. A resolution agonist, RvE1 rescues this critical impairment. In view of these findings, pro-resolving lipid mediators, such as RvE1 may be of interest for future clinical application in the treatment of inflammatory diseases like periodontitis.

## Materials and Methods

### Antibodies and Reagents

#### Antibodies

Phycoerythrin (PE)-conjugated mouse anti-human CD62P was obtained from Pharmingen (San Jose, CA) and mouse anti-human FITC-CD41 from BD Biosciences (Rockville, MD). (PE)-conjugated mouse anti-human CD18, FITC-conjugated mouse anti-human CD14, mouse anti-human CD16, Cy5-conjugated mouse anti-human CD3, and mouse anti-human CD20 were all purchased from Pharmingen (San Jose, CA). GM-CSF was obtained from R&D Systems (Minneapolis, MN), and FITC–zymosan from *Saccharomyces cerevisiae* was obtained from Molecular Probes (Carlsbad, CA). Lipoxin A_4_ ELISA was purchased from Neogen Corporation (Lexington, KY). RBC Lysis buffer was purchased from eBioscience (San Diego, CA). Histopaque 1077 and Zymosan A were purchased from Sigma-Aldrich (St. Louis, MO).

### Whole blood analysis

Human samples were obtained following informed consent under a Boston University (Boston, MA, USA) Institutional Review Board. The Boston University IRB approved our study in writing (protocol number H-23425). Venous blood (1:10 sodium citrate anticoagulant) was collected from healthy volunteers (n = 10) or patients with a diagnosis of aggressive periodontitis (LAP), n = 10 with no other known disease. All blood donors were non-smokers, between the age of 19–48 years of age who had denied taking any non steroidal anti-inflammatory drugs (NSAIDs) for at least two weeks prior to the experiment. The LAP subjects were all of African descent and were characterized by periodontal infection with multiple organisms including *Porphyromonas gingivalis* and *Aggregatibacter actinomycetemcomitans,* and a hyper-responsive neutrophil phenotype (elevated fMLP induced superoxide generation) [8]. Clinically, subjects presented with severe, early-onset bone loss around first molars and incisor teeth only [13]. The patients were diagnosed by a licensed periodontist in the Clinical Research Center of Boston University School of Dental Medicine. Healthy control donors showed no signs of periodontal disease and were matched to LAP donor based on age, sex and race.

Red blood cells were lysed using 1×RBC Lysis buffer diluted 25:1 with blood for 10 minutes on ice [55]. Direct immunofluorescence labeling was performed using anti-human CD41 and CD62P, CD16, CD14, CD20, CD3 in combination with the corresponding isotype controls to detect platelets, neutrophils, monocytes, B cells and T cells, respectively. Cells were analyzed via flow cytometry (Becton, Dickinson and Company, Franklin Lakes, NJ) and CellQuest software. Platelet-leukocyte aggregates were determined based on double positive staining for CD41 and corresponding leukocyte marker.

### Human whole blood incubations

Freshly prepared blood (1 mL, anticoagulated with Heparin) was collected and incubated with A23187 (5 µM) for 20 minutes, 37°C. Incubations were stopped on ice and plasma was immediately collected by centrifugation (350×g, 5 minutes, 4°C). Two volumes of cold methanol and deuterated internal standards (PGE_2_-d4 and 5(S)-HETE-d8) were then added. Samples were taken to C-18 solid phase extraction. Methyl formate fractions were collected, dried under nitrogen and subjected to LC-MS/MS [56].

### LC-MS/MS-based lipid mediator lipidomics

LC-MS/MS was performed with a Shimadzu LC-20AD HPLC (Shimadzu Scientific Instruments, Columbia, MD) equipped with an Agilent Eclipse Plus C18 column (4.6 mm×50 mm×1.8 µm) paired with an ABI Sciex Instruments 3200 Qtrap linear ion trap triple quadrupole mass spectrometer (Applied Biosystems, Foster City, CA). Instrument control and data acquisition were performed using AnalystTM 1.5 software (Applied Biosystems). The mobile phase consisted of methanol/water/acetic acid (60/40/0.01; v/v/v) and was ramped to 80/20/0.01 (v/v/v) after 10 min, 100/0/0.01 (v/v/v) after 12 min, and 90:10 (v/v/v) after 1.5 minutes to wash and equilibrate the column. Ion pairs from reported multiple reaction monitoring (MRM) methods [56] were used for profiling and quantification of various lipid mediators, including LTB_4_, 20-OH-LTB_4_, 5-HETE, 12-HETE, 15-HETE, and internal standards. The MRM transitions were LTB_4_ (335/195), 20-OH-LTB4 (351/195), 5-HETE (319/115), 12-HETE (319/179), 15-HETE (319/219), 14-HDHA (343/205), PGE_2_ (351/189), d8-5-HETE (327/116), and d4-PGE_2_ (355/193). The criteria used for positive identification of compounds of interest were matching retention time and matching of 6 diagnostic ions to synthetic standards. Quantification was performed using standard calibration curves for each compound, and recovery was calculated using deuterated internal standards (PGE_2_-d4 and 5(S)-HETE-d8) [56]. LXA_4_ was confirmed by LC-MS/MS and quantified by ELISA using a ThermoMax microplate reader (Molecular Devices, Sunnyvale, CA).

### Macrophage Phagocytosis of opsonized FITC-zymosan

Macrophage phagocytosis experiments were carried out as in [57]. Briefly, monocytes were isolated from human whole blood and cultured in RPMI with 10 ng/mL human GM–CSF at 37°C for 7 days. Macrophages were enumerated in a hemocytometer and viability was determined by Trypan blue exclusion. For phagocytosis of serum-opsonized zymosan A (SZ) experiments, macrophages (0.1×10^6^ cells/well in a 24-well plate) were incubated with RvE1 or vehicle for 15 min at 37°C. FITC–SZ from *Saccharomyces cerevisiae* was then added to cells (0.5×10^6^ particles/well) and incubated (30 min at 37°C) in the dark. Supernatants were aspirated, and Trypan blue (0.03% in PBS+/+ for ∼60 s) was added to quench extracellular FITC–STZ. Fluorescence was measured using a Victor plate reader (PerkinElmer).

### Data analysis

The significance of difference between groups was evaluated using the 2-tailed Student's *t-*test. P values of less than 0.05 were considered to be statistically significant.
